# Reconceptualising the study of alcohol policy decision-making: the contribution of political science

**DOI:** 10.1080/16066359.2020.1773445

**Published:** 2020-06-09

**Authors:** Matthew Lesch, Jim McCambridge

**Affiliations:** Department of Health Sciences, University of York, York, United Kingdom

**Keywords:** Alcohol policy, minimum-unit pricing, political science, Wales, decision-making

## Abstract

In this article, we demonstrate the applicability of a 3-I (interests, institutions, and ideas) framework to alcohol policy research. The analysis uses literature from political science research to provide a core theoretical framework. To help illustrate the argument, we draw on relevant examples from alcohol policy in the UK as well as initial findings from an ongoing research study on minimum-unit pricing in Wales. The Welsh case study provides an opportunity to examine the value of the framework in generating testable hypotheses in alcohol policy research. We find that several interrelated factors promoted policy change in Wales, including the government’s power to legislate on matters of public health (institutionally), a relatively weak alcohol industry (a key interest group), and a public health community with specific policy arguments on why and how to tackle alcohol-related harms (ideas). Our analysis has important implications for public health research and evidence-based policymaking. It suggests that the uptake of new ideas depends on the existing configuration of interests, institutions and ideas. This analysis provides alcohol policy researchers with a portable framework for analysing the policy context.

Reducing the harms associated with tobacco and alcohol use have been key priorities for the public health community. In the case of tobacco, efforts to strengthen international and domestic laws have been widely successful. The last two decades have witnessed major shifts in how tobacco is marketed, sold, and ultimately consumed (World Health Organisation 2017). Several interrelated factors have contributed to a global convergence of tobacco policy, particularly scientific consensus about the health risks of tobacco use, shifting public attitudes toward smoking, and advocacy from public health organisations (Cairney and Studlar 2014). Efforts to make progress on alcohol have been less successful.

The World Health Organization (2010) estimates that the harmful use of alcohol contributes to about 3.3 million deaths globally each year. Despite this evidence, governments have not responded with appropriate policy measures. Existing research suggests that alcohol industry actors’ activities have played a key role in resisting evidence-based alcohol policies (Jernigan and Trangenstein 2017; McCambridge et al. 2018). These findings reflect a broader concern about the gap between scientific evidence and policy (Lorenc et al. 2014; Oliver and Boaz 2019) and the potential role of political factors in driving this chasm (Liverani et al. 2013; Parkhurst 2017).

Engagements with distinct research traditions may help close the evidence-policy gap (Oliver and Boaz 2019). To this end, some public health researchers have turned to theoretical traditions from political science (Frohlich et al. 2004; Breton and De Leeuw 2011; Smith 2013; Smith and Katikireddi 2013; De Leeuw et al. 2014; Harris et al. 2014; Fafard 2015; Baum et al. 2018). Researchers have drawn on different approaches, including new institutionalism (Harris et al. 2019), policy subsystems (Harris et al. 2014), and policy networks (Shearer et al. 2016). Others have used the three dominant theories of the policy process – Multiple Streams Framework (MSF), Punctuated Equilibrium Theory (PET) and Advocacy Coalition Framework (ACF) (John 2003; Real-Dato 2009) – and applied these to public health policies (Breton and De Leeuw 2011; Baum et al. 2018; Harris et al. 2018). These works reflect a concerted effort to use political science (Gagnon et al. 2017) to uncover ‘the determinants of policy choice’ (De Leeuw et al. 2014, p. 3).

In this article, we look to extend this engagement by turning to an alternative framework for studying alcohol policy. Unlike previous work, the focus is not on applying theories of the policy process (Breton and De Leeuw 2011; Baum et al. 2018; Harris et al. 2018). Instead, we outline a more general framework that introduces alcohol researchers to the building blocks of political science research: interests, institutions and ideas.

The 3-I framework develops the interrelationships between 1) *interests* (i.e. actors and power), 2) *institutions* (i.e. rules, processes and context), and 3) *ideas* (i.e. beliefs and values) in explaining political phenomena. Causal explanations in political science often draw on interest-based, institutional or ideational arguments (Pontusson 1995; Hall 1997; Lieberman 2002; Hay 2004). Here we emphasise the value of recognising synergies between these factors; the interplay between interests, institutions and ideas for analytic purposes. This is not the first analysis to suggest these factors matter or are interrelated (Heclo 1994; Hall 1997). Following other health policy analysts (Lavis et al. 2002; Bashir and Ungar 2015; Shearer et al. 2016; Schram 2018), we propose that employing the 3-I framework can provide alcohol researchers with a more sophisticated understanding of how politics shapes the policy process. To our knowledge, this is the first effort to apply this framework to alcohol policy.

We begin by reviewing the secondary literature that informs the conceptual approach. In the theoretical discussion, we also point to existing empirical research to provide illustration of the 3-I framework. Next, we use preliminary findings from a case study, the adoption of minimum-unit pricing (MUP) in Wales, to further support the argument. Data for the case study is drawn from secondary literature, primary documents, and newspaper articles. Newspaper articles were collected using the Nexis database. We searched UK and Wales media sources to trace the MUP debate between 2010 and 2017. This helped construct a narrative of the case, allowing us to draw some inferences and identify testable hypotheses for future research.

## Alcohol, public health, and policy

Alcohol poses a range of public policy challenges for society. As such, there has been a concerted effort to document the global scale of alcohol’s health-related harms (Babor and Robaina 2013). The more alcohol is consumed, the more harm should be expected. Such a relationship is robustly observed across populations and for a wide range of health- and non-health related harms (Babor et al. 2010). Researchers have long recognised the importance of public policy in addressing alcohol-related harm. As Burton et al. (2017) summarise, harms from alcohol are primarily a function of a small number of key determinants. These are affordability, availability (i.e. ease of access), and social norms (i.e. acceptability). A wealth of international evidence suggests that limiting the availability (e.g. outlet density), increasing price (e.g. taxation), and to a lesser extent restricting promotion (e.g. advertising) are the most effective policy tools in curbing alcohol-related harm (Babor et al. 2010; Burton et al. 2017; World Health Organization 2019). Yet many governments have failed to adopt these policy measures, suggesting key impediments inhibit the uptake of these ideas.

The alcohol industry has a vested interest in how governments respond to alcohol-related harms (McCambridge, Hawkins, et al. 2014; McCambridge, Coleman, et al. 2019). Yet this conflict of interest is rarely acknowledged by governments (McCambridge, Kypri, et al. 2019; Hawkins and McCambridge 2020). Changes to tobacco policymaking are relevant and instructive. Tobacco companies are no longer treated as legitimate public health policy actors. Under the Framework Convention on Tobacco Control (FCTC) Article 5.3, signatory governments are forbidden from engaging with tobacco companies in designing tobacco control policies. Yet alcohol industry actors are routinely consulted and regularly form partnerships on policy priorities (Hawkins and McCambridge 2020). This might be important to understanding how industry actors exercise their power. But it is also necessary to consider how far industry influence in policymaking accounts for the speed of alcohol policy development, and what other factors may be involved. The existing alcohol policy research has not extensively theorised the policy process or tested the effectiveness of specific industry strategies on policy decision-making in particular contexts.

## Political science approaches to explaining policy development

Understanding why governments make specific policy choices about alcohol is fundamentally about power and thus requires political analysis. Alcohol researchers have used political science concepts including framing of ideas (Chong and Druckman 2007) and policy windows (Kingdon 1995) to explain alcohol policy developments. The focus of this research, however, has tended to be on the alcohol industry and its efforts (McCambridge et al. 2018). Less weight in existing analyses has been given to the influence of institutional factors in mediating or moderating the industry’s success; interest-based analyses, in particular, predominate. The remainder of this section describes each aspect of the 3-I framework as well as introduces their inter-relationships. For clarity, though, the section begins by introducing these factors individually.

### Interests

Interest-based accounts focus on actors’ policy preferences and the power to secure those aims. Organised interests tend to be the prevailing focus of these analyses. Actors recognise their common material interests and mobilise their resources to attain their preferred ends. The capacities of organised interests to achieve these goals hinge on what power they possess, including what resources they can bring to bear on those with decision-making power.

Interest-based analyses tend to focus on the disproportionate power of business in shaping policy outcomes. Corporate interests have been shown to have powerful influences on policy, especially in cases where issue salience is low (Culpepper 2010; Hacker and Pierson 2011). The reasons for this are two-fold: first, business interests enjoy disproportionate access to decision-makers (Young and Everitt 2005; Hacker and Pierson 2010). Second, elected officials have incentives to be responsive to corporate interests (Schattschneider 1960; Wilks 2013), particularly given the growing complexity of public policy issues and perennial concerns about economic growth (Fuchs and Lederer 2007).

Existing work on the alcohol industry finds that corporate actors mobilise their resources to coordinate political strategy (McCambridge, Coleman, et al. 2019). The alcohol industry is made up of several different constituent parts (i.e. drink type, producer, and retailer), meaning that policy preferences can vary depending on the policy in question (Holden et al. 2012). One common means of co-ordination is through the creation of trade associations (Holden and Hawkins 2013) and social aspect organisations (SAOs), which promote industry framing of ideas and advance shared interests (Mialon and McCambridge 2018) in other ways.

Industry actors seek to advance their interests in policy institutional contexts and in the realm of ideas more broadly. Alcohol consumption produces various externalities, including health damage to the individual and broader social consequences. Taxation, and other pricing mechanisms, can be used to address these harms, forcing producers and/or users to internalise the cost of the behavior. Yet industry actors emphasise the responsibility of the individual drinker in generating such externalities (Hawkins and Holden 2013). The goal is to avoid being seen as responsible, and thus a possible target for policymakers’ attention. Organised interests possess the resources and incentives to frame the issues under consideration by policymakers, though this is done in competition with other actors, and in the case of alcohol, these are public health actors (Katikireddi, Bond, et al. 2014).

### Institutions

In the past three decades, institutional explanations have proliferated in political science. ‘New institutionalism’ stresses the impact of formal rules, norms, and historical legacies on political behaviors and policy choices (Hall and Taylor 1996; Thelen 1999). As North (1990, p. 3) explains, institutions can be conceptualised as ‘humanly devised constraints that shape human interaction.’ A major contribution of this literature is that it specifies the mediating impact of a political system’s institutional characteristics, including the division of lawmaking authority (Weaver and Rockman 1993; Pierson 1995), on other key variables in political processes.

Institutional scholarship comprises four different approaches: Rational choice institutionalism (RI), historical institutionalism (HI), sociological institutionalism (SI), and discursive institutionalism (DI). The ontological assumptions of these traditions vary but they see institutional context as consequential for interests (RI, HI), identities (HI and SI), and ideas (SI and DI) (For reviews Hall and Taylor 1996; Schmidt 2008). We draw primarily on RI, HI and DI in the framework.

Institutional characteristics matter for policy because they determine how decision-making power is constituted and distributed (Hall and Taylor 1996; Thelen 1999). As RI approaches demonstrate, the institutional make-up of a political system determines the number of veto points in the legislative process (Tsebelis 2000). These rules of the game induce policy actors, including organised interests, to adopt specific tactics and strategies (Immergut 1990; Pierson 1996).

Institutional analyses are also needed because public policy decisions are not made in a vacuum. Following work on HI, institutional perspectives on the policy process stress the resilience of existing policy arrangements (Pierson and Weaver 1992). Based on a logic of path dependence, policies become locked-in. By virtue of history, specific actors (i.e. interests) and ways of thinking (i.e. ideas) become institutionally embedded and reinforced over time (Pierson 2000; Jacobs and Weaver 2015). At the same time, a long-standing critique of new institutionalism is its treatment of change. Institutional approaches seem better suited to explain policy stasis and less equipped to explain change (Thelen 1999; Béland 2009).

In the context of alcohol, institutional access to decision-makers has often enabled industry actors to influence the government’s agenda. If industry actors are routinely consulted about policy problems as well as potential solutions, this offers a key opportunity to protect their commercial interests. The Responsibility Deal, initiated by the UK government in 2011, offers a clear illustration of how institutional access can bias the direction of alcohol policy in the industry’s favor (Knai et al. 2015).Thus, institutional approaches are helpful in understanding some features of alcohol policy development.

### Ideas

Political scientists have also suggested that policymaking should be conceptualised as a battle of ideas (Béland and Cox 2011). Ideas can be conceptualised as something much broader than specific policy solutions; they often involve more general claims about ‘causal relationships, or the normative legitimacy of certain actions’ (Parsons 2003, p. 48). The ACF, for example, highlights the role of shared policy beliefs in driving collective action. ACF scholars posit that actors will organise into competing coalitions, where the beliefs of a winning coalition are expressed in government policy (Sabatier and Weible 2014).

Ideational scholars have theorised different causal mechanisms, stressing the interplay of ideas and institutions in promoting change. Through processes of *policy learning* (May 1992), actors will draw on new information to update their causal beliefs about the efficacy of a particular policy (Dunlop and Radaelli 2018). Ideational processes can also be less technocratic. Actors can use *ideational power* (e.g. discourse, issue framing) to shape the normative and cognitive beliefs of other key actors (Carstensen and Schmidt 2016). Finally, the arrival of new actors with new *policy paradigms* is another key source of policy change (Hall 1993; Skogstad and Schmidt 2011). These works offer clearer insights into the different mechanisms that underlie major policy change.

Ideational processes play a key role in public health policy processes, including alcohol policy (Schram 2018). For example, one idea perpetuated by industry actors is that while alcohol can be harmful, this concern is restricted to a narrow set of problem-drinkers (Bond et al. 2009; McCambridge et al. 2018). This is at odds with the evidence (Babor et al. 2010; Burton et al. 2017; World Health Organization 2019). Industry actors seek to dominate the information environment within policy-making, framing key ideas in particular ways, so as to marginalise the scientific evidence (McCambridge, Kypri, et al. 2014). Industry actors routinely contest the interpretation and use of scientific findings in policymaking (Rossow and McCambridge 2019), and indeed ideas about the relationships between science and policy (McCambridge, Daube, et al. 2019). It is noteworthy that in countries where major policy change has been achieved, industry efforts to misrepresent scientific evidence have not succeeded (McCambridge et al. 2013).

### Integrating interests, ideas, and institutions

Policy dynamics should be understood as resulting from interactions, including frictions, between institutional, ideational and interest-based factors (Lieberman 2002; Béland 2009). Theories of the policy process typically imply these interactions but do not explicitly incorporate these dynamics into their models. As Real-Dato (2009, p. 119) argues, the dominant policy process theories have not come to grips with new institutionalism, providing ‘limited treatment’ of institutional constraints. We see synergies between policy process theories and the 3-I approach, particularly as it relates to the institutions. For example, in the Punctuated Equilibrium Theory (PET), the concepts of *policy monopolies* and *policy images* (Baumgartner and Jones 1993) capture the interrelationships identified by the 3-I framework.

Policy monopolies refer to closed models of policymaking, where a defined set of policy actors possess *de facto* control over a policy area. High levels of policy stability can be attained by locking-in who participates, as well as what policy ideas are contested and considered legitimate (Baumgartner et al. 2009). One of the specific ways in which control is exerted within a policy monopoly is due to participants’ shared understanding of a policy problem. Policy images refer to ‘how a policy is understood and discussed.’ When a single image of a policy area is widely accepted, it is remarkably resistant to policy change (Baumgartner and Jones 1993, p. 25). But policy monopolies and policy images can also be dislodged via new ideas and new actors (Hall 1993). Those excluded from the policy monopoly often recognise the impenetrability of existing decision-making structures and seek out alternative institutional contexts that may be more responsive to their goals. These contexts provide those on the losing side (Baumgartner and Jones 1993; Pralle 2003) with alternative venues to help reframe the policy image serving the existing policy monopoly. As scholarship in new institutionalism reminds us, however, the capacity to access these alternative institutions will be shaped by the internal developments of those structures. The capacity to shift policy images and/or venue shift will be circumscribed by the institutional context of new settings.

The employment of such concepts is fruitful for understanding how specific alcohol policy measures are kept off government agendas. Institutionalised policy practices, such as government-industry partnerships, can preclude meaningful discussion of policy ideas defined as threatening to the industry’s interests. Under such conditions, industry actors may not depend on lobbying, or other overt attempts to influence elected officials so long prevailing ideas or policy images prevail. This has implications for how researchers study industry influence. Rather than looking only at donations or lobbying, attention could be paid to how industry actors shape how elected officials define alcohol as a policy problem. Finally, as illustrated below, new policy ideas with a strong evidence base can be advanced by a coalition of civil society actors.

Critically, the 3-I framework is not the only way to conceptualise the policy process. As noted above, health policy researchers have already begun to use policy process theories (Breton and De Leeuw 2011; De Leeuw et al. 2014; Fafard 2015; Baum et al. 2019). These approaches have a lot to offer (see Baum et al. 2019). They provide compelling insights into the role of shared beliefs (ACF), policy windows (MSF), and venue shifts (PET) in shaping policy dynamics. One potential limitation of employing these frameworks is that they require specification of scope conditions (Schlager 2007). For example, the MSF is helpful for understanding how issues get on the agenda but is limited in explaining political dynamics that unfold during policy implementation (though for a recent application to alcohol policy see Hawkins and McCambridge 2020). The ACF is interested in explaining policy change but over an extended period of time (i.e. 10 years or more). This means this approach is less attentive to the immediate circumstances around adoption of specific policy measures (Schalger 2007), such as MUP in Wales. A key advantage of the 3-I framework is that it is portable across different stages of the policy process, making fewer assumptions in so doing. Moreover, by providing conceptual clarification at a higher level it enables analysts to approach the study of the policy process more flexibly. The framework, then, might be particularly instructive when researchers are mapping out the core features of the case and not testing specific hypotheses derived from a particular policy process theory.

Another approach surveys the characteristics of policy subsystems (Howlett et al. 2009), examining the role of *actors* (i.e. roles, values and networks), *institutions* (i.e. different structural factors) and *ideas* (i.e. the content of policy) (Harris et al. 2014, 2018). The benefit of the policy subsystems approach is that it can enable analysts to apply multiple policy process theories to the same case (Harris et al. 2018). There is considerable overlap with this approach and the 3-I framework. In the 3-I framework, however, interests and actors are not treated as synonymous; actors are taken as relevant across all three categories. A second difference is the treatment of ideas. In the 3-I framework (but also see Harris et al. 2018), ideational processes are treated as broader than the explicit content of policy solutions (Campbell 1998; Mehta 2010).

## The case of minimum unit pricing in Wales

We now draw on initial findings from an ongoing research study on minimum-unit pricing in Wales to illustrate how this conceptual work could be applied as well as inform future research.

The Public Health (Minimum Price for Alcohol) (Wales) Bill was passed into law in June 2018. The decision to adopt MUP is examined here to provide an illustration of how ideas, interests, and institutions may operate and intersect in the policy process.

Alcohol has been a long-standing public health concern in Wales. Welsh policymakers have long been urged to tackle various alcohol-related harms, including health issues, public disorder and domestic violence (Government of Wales 2017). Over the past forty years, Wales has seen a steady rise in alcohol-related deaths and hospital admissions. Alcohol imposes a major financial burden on public finances, with a recent estimate of £76.5 million per year (Government of Wales 2017). Addressing these harms, as well as other unhealthy behaviors, has thus formed a key policy priority since power was devolved to the WNA in 1999 (Porter and Miloudi 2009).

Access to inexpensive alcohol has been identified as a key culprit in driving alcohol-related harm. Research has demonstrated a strong link between cheap alcohol and harmful levels of drinking (Stockwell et al. 2012). Governments across the UK, including Wales, have looked for ways to reduce consumption of low-cost and high-alcohol content products (Government of Wales 2017). Research suggests that increasing the cost of alcohol (i.e. taxing it) can reduce consumption (Babor et al. 2010). In the UK, however, taxation is a power reserved for Westminster.

Governments have other alternatives beyond taxation to increase the cost of alcohol. In 2008 public health advocates identified MUP as a potential way to reduce alcohol-related harm. For the UK’s devolved administrations, the instrument was particularly attractive. MUP imposes a price floor but does not impose a tax and thus would likely fall under these governments’ legislative competence.

MUP first gained significant traction within Scotland. In 2012, the Scottish Parliament passed the Alcohol (Minimum Pricing) Scotland Act 2012 (Katikireddi, Hilton et al. 2014). A legal challenge by the Scottish Whiskey Association (SWA) quickly followed, claiming the policy breached EU competition law (Katikireddi, Bond, et al. 2014). Although MUP was eventually upheld, the legal challenge significantly delayed implementation to May 2019.

An important consequence of the policy debate in Scotland is that it drew the interest of other jurisdictions. In 2012, as part of its Alcohol Strategy, the UK government announced its intention to bring in MUP, a policy that would apply to England and Wales. Facing industry resistance, however, the UK government shelved these plans the following year (Gornall 2014; Hawkins and McCambridge 2014; Nicholls and Greenaway 2015; Gornall 2014). For the Welsh government, however, Westminster’s U-turn on MUP presented an opportunity to develop its own MUP (Rutherford 2014).

In the start of 2014, the Welsh government asked its Advisory Panel on Substance Misuse (APoSM) to review the literature on alcohol pricing. The advisory panel reported back to the government, describing the evidence in support of MUP as both ‘extensive and reliable’ (Government of Wales 2017, p. 10).

By April 2014, the government launched a Public Health White Paper. MUP was described as a ‘proportionate and preventative action [that could] protect *public health’* (Government of Wales 2014). The government defended the policy referring to the University of Sheffield’s research on consumption and price (‘Alcohol pricing plans shelved,’ 2013). It then commissioned these researchers to model the impact of different pricing scenarios on various outcomes (Government of Wales 2014). The results suggested that the introduction of 50p could save the Welsh treasury £882 million annually by significantly reducing illness, crime, and workplace absenteeism (Meng et al. 2014).

Opposition to both the draft bill in 2015 and legislation in 2017 was remarkably muted. There were some objections raised to the bill. Alcohol producers and retailers provided written submissions and offered testimony during the committee stage of the bill. In comparison to the MUP debate in Scotland and England, however, the alcohol industry seemed far less engaged. One key difference might be the scale of alcohol production, which is much smaller in Wales (National Assembly for Wales Rural Development Sub-Committee 2010). Of the industry groups which opposed the Welsh legislation, most were London-based alcohol producers, retailers or trade associations (National Assembly for Wales Health 2018). Finally, in contrast to Scotland, MUP did not divide the major political parties. Only 5 out of 45 assembly members voted against the legislation in Wales, with UKIP being the only party to oppose the measure (Jones 2017).

### The role of interests, institutions and ideas in the development of MUP in Wales

One of the more striking things about this case is the relative absence of the alcohol industry in policymaking. In comparison to MUP reform in Scotland (Holden and Hawkins 2013) alcohol industry groups played a much less prominent role in the Welsh debate. One potential way to understand the industry’s limited presence is in the broader institutional and ideational context. The Welsh Assembly lacks many of the legislative powers other than health that are directly related to the interests of alcohol industry actors (e.g. taxation, trade, and criminal justice). Such institutional conditions can shape the political organisation of interest groups.

In the UK, industry actors have embedded themselves into the policy context at Westminster (McCambridge, Hawkins, et al. 2014). Based on the 3-I framework, we hypothesise that the alcohol industry was less prominent in the Welsh case because they lacked access to key decision-makers and departments. In contrast to Westminster, the Welsh government had never previously legislated on alcohol-specific matters. This suggests there were fewer reasons for the industry to engage directly with the Welsh government. Additional data sources will be needed to further investigate the nature of industry activity in Wales, and the extent to which it was shaped by such institutional factors rather than by the nature of the industry there.

Second, beyond these strategic imperatives, the institutional context can also shape how government actors define policy problems and solutions, or the prevailing policy image. In Wales, responsibility for public health is devolved, while crime and licensing are not. This led the government, eventually, to adopt the MUP legislation (Connell 2019). In the Westminster institutional context, however, a public health framing of alcohol is forced to compete with other potential framings. A second direction for research is that devolved responsibility for public health provided favorable political conditions for public health advocacy and framing of alcohol-related harms.

Extending this line of thinking, broader institutional forces can also be seen to have shaped the trajectory of MUP in the Welsh context. Devolution has resulted in greater opportunities for policy innovation, providing the policymaking authority and fiscal capacity to experiment with new policies. In doing so, devolution also facilitates policy transfer across the UK (Cairney 2007, 2009). Is MUP a case of policy transfer in the UK, then? Scotland’s interest in introducing MUP preceded policy discussions in Wales. In the Welsh government’s 173-page explanatory memorandum on MUP, the Scottish policy or context is referred to 34 times (Government of Wales 2017). Dedicated research will be needed to investigate how far Welsh adoption of MUP can be considered a case of policy transfer. Particular attention needs to be paid to the underlying mechanism in so doing. For example, did decision-makers in Wales learn from their counterparts in Scotland? Or did they simply emulate the Scottish MUP policy design?

Finally, the idea of MUP itself pervades much of this case. Two expert groups, the Advisory Panel on Substance Misuse and University of Sheffield researchers, played key roles in helping formulate MUP. The former group helped frame the nature of the policy problem, while the latter offered recommendations for designing the policy instrument. Clearly, these experts played a key part in the formal justification of MUP. What is less clear from the existing data, however, is whether, and if so how and why the work of these experts persuaded elected officials to ultimately support MUP. Ongoing study of Wales will test the robustness of these preliminary conceptually informed observations, refine the research questions, and may shed new insights into the nature of alcohol policy decision-making processes, both in Wales and elsewhere.

## Conclusion

Public health researchers have long been frustrated by policy inertia. There has been growing interest in using political sciences approaches to help better understand the causes and consequences of such inertia (De Leeuw et al. 2014; Mackenbach 2014). In this article, we contribute to this effort by clarifying and then illustrating how a 3-I (interests, institutions, and ideas) framework can be used to study alcohol policy decision-making. The framework provides a portable set of tools that can be used to study alcohol policy developments as well as other policy issues in public health. Research funders have a key role to play in facilitating this endeavor.

The framework does not offer specific recommendations for overcoming the political barriers to policy change. It does, however, hold some important implications. One clear implication is that reform advocates should choose their battles wisely. As institutional theory suggests, existing policy arrangements are highly resistant to change (Thelen 1999). Rather than attempting to persuade decision-makers and/or opponents with evidence and argumentation, advocates might consider a different tactic. Advocates should attempt to grow their coalition by broadening the appeal of their preferred policy interventions through re-framing (Mehta 2010). Coalition-building can help undermine the dominance of a prevailing discourse (Schmidt 2008) or policy image (Baumgartner and Jones 1993).

At its core, political science is fundamentally concerned with understanding the shape, distribution, and exercise of political power (Arts and Tatenhove 2004; Hay 1997; Lasswell and Kaplan 2017). If the ultimate aim of public health research is to inform the content of policy then researchers require a clearer conceptualisation of how power functions in the policy process. The political context needs to be properly understood in order for power to be used effectively in the interests of public health. This article has clarified the range of analytic tools that can be deployed for such purposes.
